# A Simple Method for the Accurate Volumetric Measurement of Superficial Tumours

**DOI:** 10.1038/bjc.1965.39

**Published:** 1965-06

**Authors:** E. J. Delorme


### A SIMPLE METHOD FOR THE ACCURATE VOLUMETRIC MEASUREMENT OF SUPERFICIAL TUMOURS

E. J. DELORME*

*From the Chester Beatty Research Institute, Institute of Cancer Research, Royal Cancer Hospital, London, S.W.3*

Received for publication December 18, 1964

PREVIOUSLY described methods of determining exact size and growth rate of tumours in living animals possess disadvantages of imprecision in the measuring tool which is usually unsuited for use on soft tissues, or dependence upon subjective assessment. Older clinical terms so frequently founded on quaint analogy : " millet seed ", " hazel nut ", " tangerine orange ", etc., obviously have no place in the quantitative investigation of tumours.

Attempts to improve tumour measurement techniques have been made by Stock and Sugiura (1957) who used successive tracings of tumour outlines on transparent paper to obtain planimetric values. Vofkori (1963) has described a more accurate but expensive and time-consuming method : that of preparing plaster casts of tumours at successive stages of growth. The method at present in most general use in laboratories and clinics is the caliper measurement of cross-diameters with or without the extrapolation of these values into a volumetric conversion employing the well-known formula based on the radius of a sphere (Schrek, 1935). This latter method depends for its accuracy upon the assumption that tumours are perfect spheres which is seldom if ever the case. Irregular, lobed or flattened tumours do not lend themselves to this volumetric derivation from diameters, and tumour mass data so derived may be grossly misleading.

A simple apparatus has been devised in this laboratory which employes the plethysmograph modified for surface application. It consists of a wide-mouthed dome-shaped vessel of a rigid material with the mouth sealed by a loose diaphragm of thin rubber. This rubber membrane must be firmly bonded to the lips of the cup by a water-tight sealing material such as the epoxy-resin adhesive (" Araldite "). The dome of the cup is connected by tubing to an upright 25 ml. calibrated pipette. A quick release clip is fitted to the tubing. The cup and tubing are filled with water which reaches the lower part of the pipette scale.

A base-line reading, A (Fig. 1), is first obtained by pressing the diaphragm firmly against a flat surface or against the clipped animal on the side opposite to the tumour and noting the water level in the pipette. The latter method is preferable because it corrects for changes in skin thickness and subcutaneous fat. The volume of the tumour may then be obtained by pressing the diaphragm against the tumour and taking a second reading, B, the difference between A and B being the volume of the tumour in cubic millilitres or fractions thereof.

The clip on the tubing is used to save time by preventing the level of water in the pipette falling between readings A and B.

Measurements may be taken rapidly on a large number of animals with minimal disturbance and handling. It may be necessary to have two or three sizes of cups

* Medical Research Council external staff.

for use with different species or for tumours of widely varying size but most experimental tumours of ordinary dimensions can be measured by one instrument with a scale of 0·1 to 25 ml. The only skin area in which the readings may be difficult is over the thin, yielding abdominal wall of small animals. For the smallest tumours a thimble-sized cup attached directly to a vertical capillary tube is all that is required.

FIG. 1.

An essential point in the construction of this instrument is that the diaphragm be fitted without tension to ensure accurate molding to the tumour and to avoid errors due to " tenting ". Folding of the loose diaphragm during zero readings does not significantly affect the accuracy of the reading. Correlation is high between instrument values and actual volumes of plasticine models of tumours.

SUMMARY

In evaluation of therapy on tumour growth rate in the intact animal, often the least accurate parameter is the measurement of tumour size. The method of mensuration described is inexpensive, time-saving and accurate to about 0·2 ml. It can be adapted to the volumetric measurement of most surface swellings in animal or man.

### REFERENCES

SCHREK, R.—(1935) *Amer. J. Cancer*, **24**, 807.
STOCK, C. AND SUGIURA, C.—(1957) *Acta Un. int. Cancr.*, **11**, 186.
VOFKORI, J.—(1963) *Neoplasma*, **10**, 187.


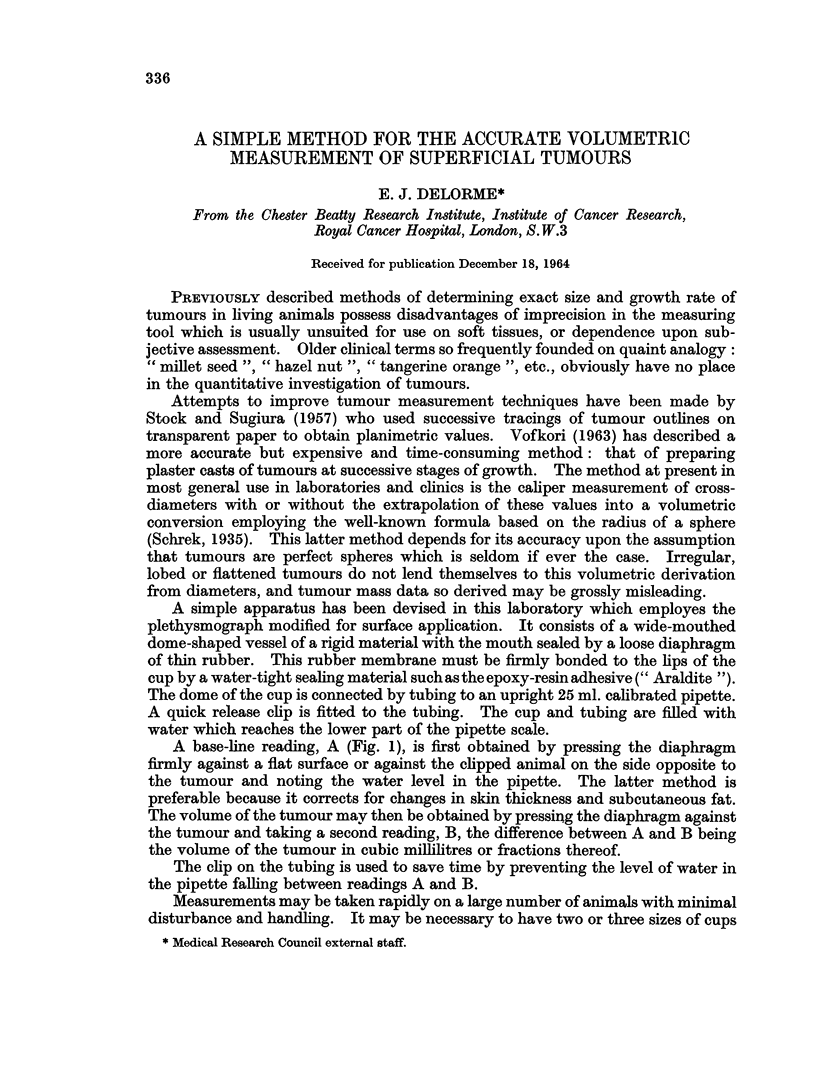

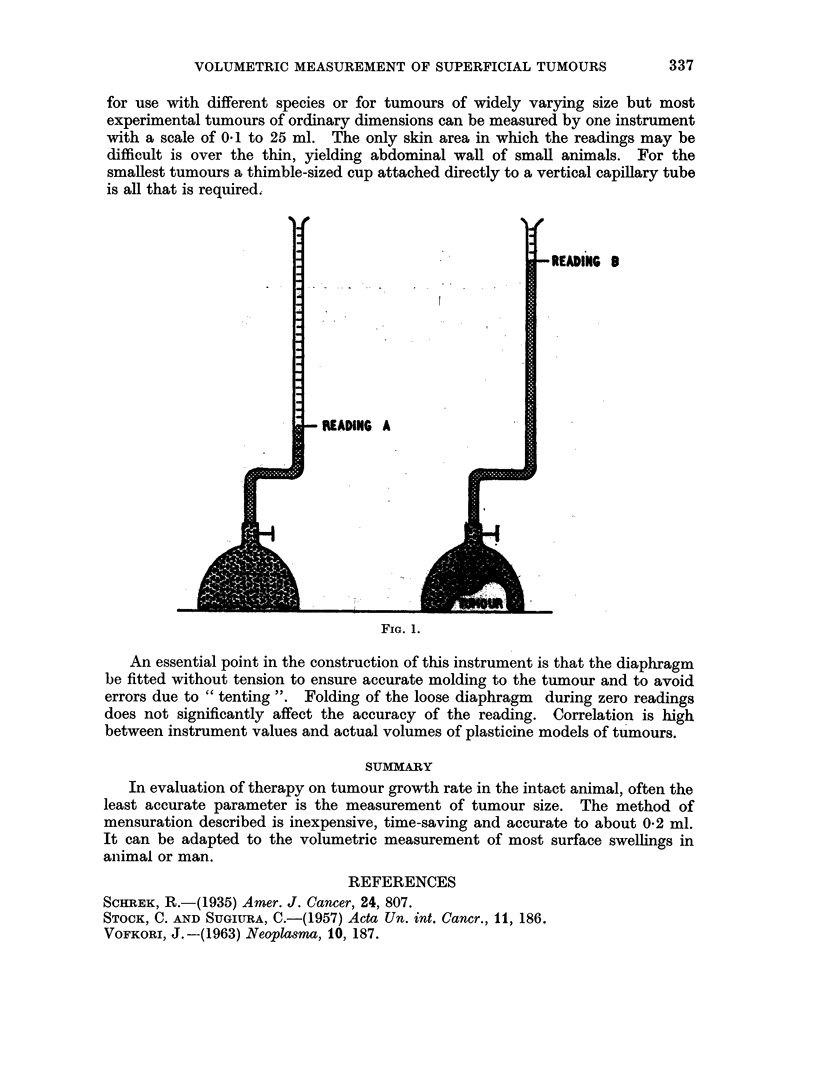


## References

[OCR_00096] VOFKORI J. (1963). Method for the measurement of the size of transplantable tumours of rats.. Neoplasma.

